# Bile acid profiles in adult patients with biliary atresia who achieve native liver survival after portoenterostomy

**DOI:** 10.1038/s41598-024-52969-6

**Published:** 2024-01-30

**Authors:** Masahiro Takeda, Hajime Takei, Mitsuyoshi Suzuki, Takafumi Tsukui, Koichi Tsuboi, Hiroko Watayo, Takanori Ochi, Hiroyuki Koga, Hiroshi Nittono, Atsuyuki Yamataka

**Affiliations:** 1 Department of Pediatric Surgery, Juntendo University School of Medicine, 2-1-1 Hongo, Bunkyo-ku, Tokyo 113-8421 Japan; 2 Junshin Clinic Bile Acid Institute, Tokyo, Japan; 3 Department of Pediatrics, Juntendo University School of Medicine, Tokyo, Japan

**Keywords:** Cholestasis, Liver cirrhosis, Liver fibrosis

## Abstract

Bile acids have received increasing attention as a marker of the long-term prognosis and a potential therapeutic target in patients with biliary atresia, which is a progressive disease of the hepatobiliary system. A detailed analysis of serum and urinary bile acid compositions was conducted to assess the characteristics of bile acid profiles and the correlation between bile acid profiles and liver fibrosis markers in adult patients with biliary atresia who achieved bilirubin normalization. Serum total bile acids and glucuronide-conjugated (glyco- and tauro-) cholic acids (GCA and TCA) and chenodeoxycholic acids (GCDCA and TCDCA) were significantly higher in patients with biliary atresia than in healthy controls, whereas unconjugated CA and CDCA showed no significant difference. There were no significant differences in CA to CDCA ratios and glycine-to-taurine-conjugated ratios. Urinary glycocholic acid 3-sulfate (GCA-3S) was significantly higher in patients with biliary atresia. Serum GCDCA showed a strong positive correlation with Mac-2 binding protein glycosylation isomer (M2BPGi). These results demonstrate that bile acid congestion persists into adulthood in patients with biliary atresia, even after cholestasis has completely improved after Kasai portoenterostomy. These fundamental data on bile acid profiles also suggest the potential value of investigating bile acid profiles in patients with biliary atresia.

## Introduction

Biliary atresia is a progressive disease of the hepatobiliary system and one of the most common causes of pediatric liver transplantation^1^. In recent years, bile acids have received increasing attention as a marker of the long-term prognosis and a potential therapeutic target in patients who have achieved normalized bilirubin levels after Kasai portoenterostomy^2,3^. Currently, there are two ongoing international investigational studies exploring the efficacy of intestinal bile acid transporter inhibitors in patients with biliary atresia after Kasai portoenterostomy^4^. Intestinal bile acid transporter inhibitors such as odevixibat have been approved as treatments for Alagille syndrome and progressive familial intrahepatic cholestasis (PFIC)^5^. If bile acid toxicity is the cause of long-term liver injury, it has been suggested that long-term liver damage and fibrosis may be avoided if cholestasis improves after Kasai portoenterostomy and chronic bile acid toxicity disorders are prevented by interrupting the enterohepatic cycle of bile acids^6^.

To determine why bile acid itself may be a long-term prognostic marker and its potential role in the mechanism of liver injury in biliary atresia, a comprehensive examination of bile acid compositions in long-term native liver survivors is required. The validation of bile acid profiles in adults with cirrhosis has demonstrated a strong correlation between 6-month mortality, portal hypertension and bile acid compositions, such as glycochenodeoxycholic acid (GCDCA) or taurochenodeoxycholic acid (TCDCA)^7,8^. In the validation of bile acid profiles in biliary atresia, it is crucial to focus on cases that meet the following criteria: (1) achieving normalized bilirubin levels to eliminate the impact of cholestasis on bile acid compositions and (2) studying cases in which physiological changes in bile acids have been completed, typically involving individuals aged 11 years and older^9^. Furthermore, focusing on adults who are at a higher risk for the development of liver cirrhosis due to long-term progression, it is possible to examine the relationship between bile acid profiles and liver fibrosis in long-term native liver survivors of biliary atresia.

The primary objective of this study was to assess the physiological status and characteristics of bile acid profiles in adult patients with biliary atresia and compare them with those healthy controls. The preliminary data obtained from a comprehensive analysis of bile acid profiles in serum and urine were intended to evaluate whether there is value in the further investigation of bile acids in the context of biliary atresia. As a secondary objective, we explored the potential relationship between bile acid profiles and liver fibrosis using non-invasive liver fibrosis markers in long-term native liver survivors of biliary atresia.

## Results

There were no significant differences in age or body size between patients with biliary atresia and healthy control subjects (Table 1). Notably, in these patients, despite normal bilirubin levels, clinical indicators suggestive of mild liver fibrosis were observed, such as irregular liver surface and edge on ultrasonography in 70.0%, splenomegaly in 60.0%, thrombocytopenia in 50.0%, and varices in 30.0%.

**Table 1:** Subjects at bile acid profiles measurement.

	Biliary atresia patients (n = 10)	Healthy controls (n = 10)	*P* value
Age (years)	22.2 (20.9–25.2)	23.5 (22.0–24.0)	0.52
Female:male	6:4	5:5	0.68
Height (cm)	156.1 (154.6–165.8)	164.5 (158.0–169.0)	0.17
Body weight (kg)	58.2 (51.4–64.7)	54.5 (53.3–64.3)	0.55
Body mass index (BMI)	22.8 (21.2–24.4)	21.6 (19.2–22.5)	0.16
Total bilirubin (mg/dL)	0.93 (0.86–1.13)	0.79 (0.56–0.92)	0.05
Direct bilirubin (mg/dL)	0.07 (0.05–0.10)	0.06 (0.05–0.10)	0.97
AST (IU/L)	25.0 (20.5–32.0)	19.5 (17.3–20.8)	0.01
ALT (IU/L)	21.5 (15.8–36.8)	10.5 (7.5–18.0)	0.004
GGT (IU/L)	46.5 (27.5–77.3)	15.5 (11.5–18.3)	0.02
Platelet counts (× 10^3^/μL)	111.0 (85.5–180.3)	257.0 (232.0–271.3)	0.002
Thrombocytopenia (less than 150.0 × 10^3^/μL) (n, %)	5 (50.0)	0 (0)	0.03
Varices (n, %)	3 (30.0)	–	
Child–Pugh score	5 (5–5)	–	
MELD score	7.0 (6.3–7.8)	–	
Ultrasonographic features			
Liver surface and edge: irregular and blunted (n, %)	7 (70.0)	–	
Liver parenchyma echogenicity: coarse (n, %)	6 (60.0)	–	
Right lobe atrophy (n, %)	2 (20.0)	–	
Splenomegaly (n, %)	6 (60.0)	–	

Values are presented as the median; values in brackets represent the interquartile range (IQR).

*AST* aspartate aminotransferase, *ALT* alanine aminotransferase, *GGT* γ-glutamyltransferase,* MELD* model for end-stage liver disease.

### Serum bile acid profiles

**Figure 1:**
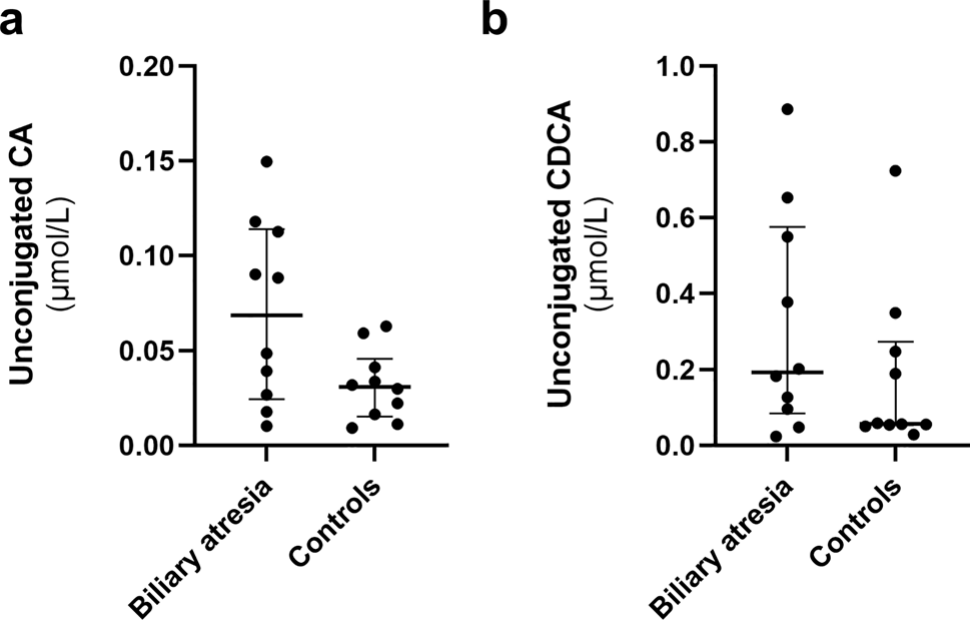
Serum unconjugated CA and CDCA in patients with biliary atresia and healthy controls. Serum unconjugated CA and CDCA levels showed no significant difference (Fig. 1a, b,  *P* = 0.09 and 0.34, respectively). The bottom line represents the first quartile, the middle line represents the median (second quartile), and the top line represents the third quartile.* CA* cholic acid, *CDCA* chenodeoxycholic acid.

**Figure 2:**
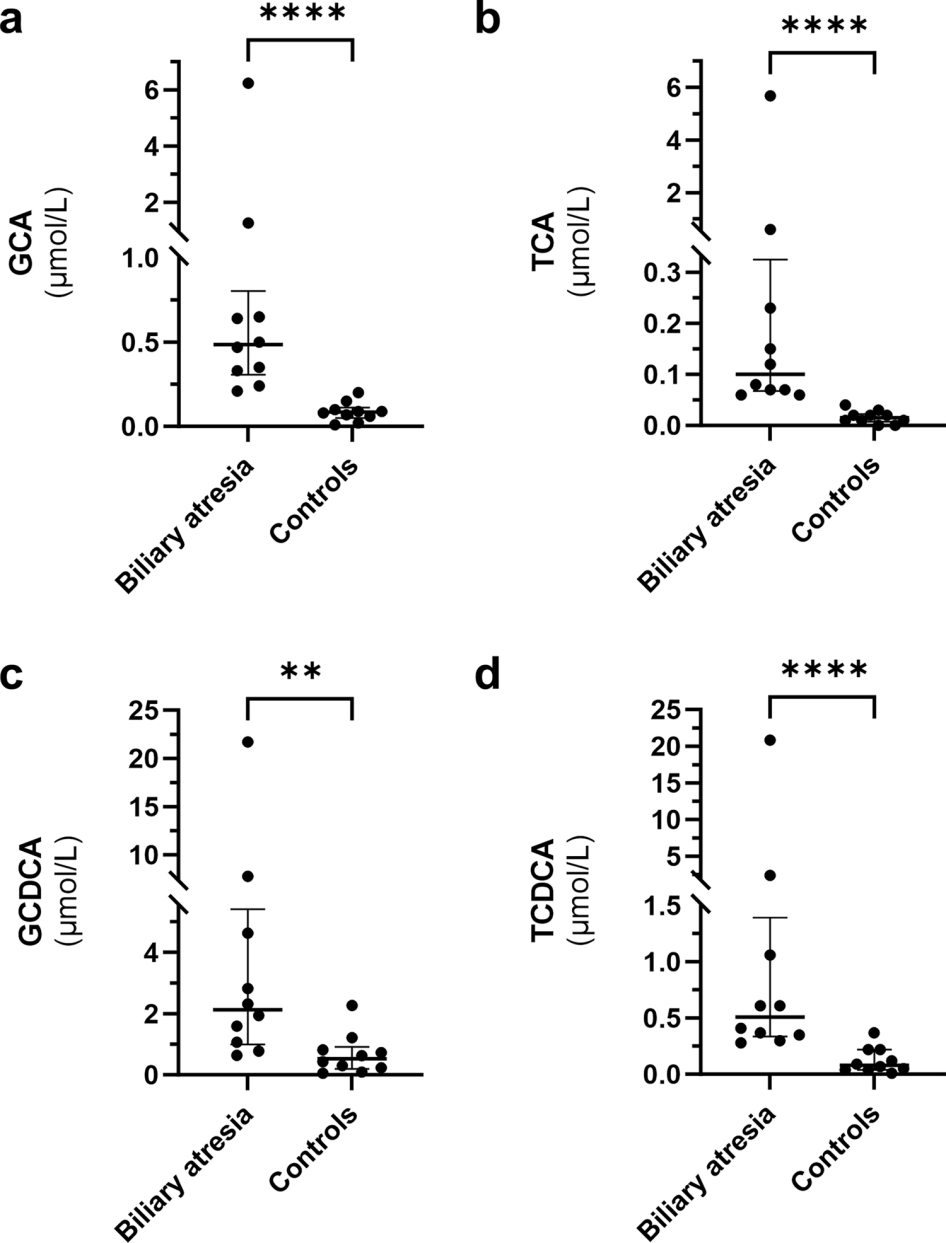
Serum glucuronide-conjugated CA and CDCA in patients with biliary atresia and healthy controls. The GCA, TCA, GCDCA, and TCDCA levels were significantly higher in patients with biliary atresia (Fig. 2a–d, *P *< 0.001, *P*  < 0.001, *P*  = 0.004, and *P*  < 0.001, respectively). The bottom line represents the first quartile, the middle line represents the median (second quartile), and the top line represents the third quartile.* CA* cholic acid, *CDCA* chenodeoxycholic acid, *GCA* glycocholic acid, *TCA* taurocholic acid, *GCDCA* glycochenodeoxycholic acid, *TCDCA* taurochenodeoxycholic acid.

In the serum bile acid profiles, total bile acids and total bile acids, excluding ursodeoxycholic acid (UDCA), were significantly higher in patients with biliary atresia (*P* = 0.004 and 0.007, respectively). The serum levels of primary bile acids [cholic acid (CA) and chenodeoxycholic acid (CDCA)] were significantly higher in patients with biliary atresia (median CA [unconjugated and glucuronide-conjugated] 0.63 μmol/L, interquartile range [IQR: 0.51–0.83] vs. 0.13 μmol/L [IQR: 0.11–0.17], *P* < 0.001 and median CDCA [unconjugated and glucuronide-conjugated] 2.86 μmol/L [IQR: 1.62–6.62] vs. 0.73 μmol/L [IQR: 0.37–1.42], *P* = 0.003). Glucuronide-conjugated (glyco- and tauro-) CA and CDCA were significantly higher in biliary atresia patients (median conjugated CA 0.57 μmol/L [IQR: 0.43–0.77] vs. 0.11 μmol/L [IQR: 0.08–0.11], *P* < 0.001 and median conjugated CDCA 2.60 μmol/L [IQR: 1.53–6.03] vs. 0.61 μmol/L [IQR: 0.31–0.98], *P* = 0.002), whereas unconjugated CA and CDCA showed no significant difference (*P* = 0.09 and 0.34, respectively) (Table 2) (Fig. 1). Glyco-CA (GCA), tauro-CA (TCA), glyco-CDCA (GCDCA), and tauro-CDCA (TCDCA) were significantly higher in patients with biliary atresia (*P* < 0.001, *P* < 0.001, *P* = 0.004, and *P* < 0.001, respectively) (Fig. 2). An additional analysis, excluding outliers in patients with biliary atresia, also showed a significant difference in glucuronide-conjugated CA and CDCA (Supplementary Fig. 1). No significant differences were observed in CA to CDCA ratios or glycine-to-taurine-conjugated ratios (Table 2). Serum sulfate-conjugated bile acids were extremely low in both groups and were excluded from further analysis (Supplementary Table 1). The bile acid profiles of patients with biliary atresia showed no significant difference between those treated with UDCA and those who did not receive UDCA (Supplementary Table 2). Serum secondary bile acids [deoxycholic acid (DCA), lithocholic acid (LCA), and hyocholic acid (HCA)] excluding UDCA were significantly lower in patients with biliary atresia (median serum secondary bile acids [unconjugated and conjugated DCA, LCA, and HCA] 0.24 μmol/L [IQR: 0–0.45] vs. 0.70 μmol/L [IQR: 0.47–0.78], *P* = 0.04). DCA was significantly lower in patients with biliary atresia (*P* = 0.002). The secondary bile acid profiles of patients with biliary atresia did not show significant differences, regardless of their history of cholangitis (Supplementary Table 3).

**Table 2:** Serum bile acids in each group.

Bile acid species	Biliary atresia patients	Healthy controls	*P* value
Total (μmol/L)	7.76 (6.04–21.5)	2.30 (1.53–4.02)	0.004
Total excluding UDCA (μmol/L)	5.07 (3.84–10.68)	2.13 (1.37–3.37)	0.007
Primary			
Unconjugated (μmol/L)			
CA	0.07 (0.03–0.11)	0.03 (0.02–0.04)	0.09
CDCA	0.19 (0.10–0.51)	0.06 (0.05–0.23)	0.34
Conjugated (μmol/L)			
GCA	0.49 (0.34–0.65)	0.09 (0.06–0.10)	< 0.001
TCA	0.10 (0.07–0.21)	0.02 (0.01–0.02)	< 0.001
GCDCA	2.13 (1.20–4.17)	0.53 (0.25–0.80)	0.004
TCDCA	0.51 (0.36–0.95)	0.08 (0.04–0.20)	< 0.001
Ratios of CA to CDCA	0.28 (0.20–0.31)	0.18 (0.10–0.29)	0.21
Ratios of glycine-to-taurine-conjugated			
CA	3.53 (1.97–6.69)	5.73 (4.99–6.34)	0.23
CDCA	3.51 (1.65–6.06)	5.11 (3.44–6.01)	0.21
Secondary			
Unconjugated (μmol/L)			
DCA	0.05 (0–0.12)	0.41 (0.25–0.51)	0.002
LCA	0.00 (0.00–0.00)	0.01 (0.00–0.01)	0.13
HCA	0.00 (0.00–0.00)	0.00 (0.00–0.00)	0.73
Conjugated (μmol/L)			
GDCA	0.08 (0–0.24)	0.20 (0.08–0.25)	0.35
TDCA	0.03 (0–0.10)	0.03 (0.01–0.04)	0.88
GLCA	0.00 (0.00–0.00)	0.00 (0.00–0.02)	0.67
TLCA	0.00 (0.00–0.00)	0.00 (0.00–0.00)	0.73
GHCA	0.01 (0–0.04)	0.01 (0.00–0.02)	0.35
THCA	0.00 (0.00–0.00)	0.00 (0.00–0.00)	0.73

Values are presented as the median; values in brackets represent the interquartile range (IQR).

*UDCA* ursodeoxycholic acid, *CA* cholic acid, *CDCA* chenodeoxycholic acid, *GCA* glycocholic acid, *TCA* taurocholic acid, *GCDCA* glycochenodeoxycholic acid, *TCDCA* taurochenodeoxycholic acid, *DCA* deoxycholic acid, *LCA* lithocholic acid, *HCA* hyocholic acid, *GDCA* glycodeoxycholic acid, *TDCA* taurodeoxycholic acid, *GLCA* glycolithocholic acid, *TLCA* taurolithocholic acid, *GHCA* glycohyocholic acid, *THCA* taurohyocholic acid.

### Urinary bile acid profiles

In the urinary bile acid profiles, total bile acid levels were significantly higher in patients with biliary atresia (*P* = 0.007), but there was no significant difference in total bile acids excluding UDCA (*P* = 0.47) (Table 3). There was no significant difference in primary bile acid levels between patients with biliary atresia and healthy controls [median CA (unconjugated, glucuronide-, and sulfate-conjugated) 0.01 μmol/L [IQR: 0.01–0.03] vs. 0.01 μmol/L [IQR: 0.01–0.02], *P* = 0.73 and median CDCA [unconjugated, glucuronide-, and sulfate-conjugated] 0.07 μmol/L [IQR: 0.01–0.14] vs. 0.01 μmol/L [IQR: 0.00–0.03], *P* = 0.08]. Glycocholic acid 3-sulfate (GCA-3S) was significantly higher in patients with biliary atresia (*P* = 0.04). The levels of unconjugated and glucuronide-conjugated bile acids in both groups did not differ significantly. Secondary bile acids were extremely low in both groups and excluded from subsequent analyses (Supplementary Table 4).

**Table 3:** Urinary bile acids in each group.

Bile acids species	Biliary atresia patients	Healthy controls	*P* value
Total (μmol/L)	1.94 (1.33–2.82)	0.44 (0.25–0.56)	0.007
Total excluding UDCA (μmol/L)	0.36 (0.21–0.63)	0.29 (0.12–0.47)	0.47
Primary			
Unconjugated (μmol/L)			
CA	0.00 (0.00–0.00)	0.00 (0.00–0.01)	0.17
CDCA	–	–	
Conjugated (μmol/L)			
GCA	0.01 (0.00–0.01)	0.00 (0.00–0.01)	0.47
TCA	0.00 (0.00–0.00)	0.00 (0.00–0.00)	0.76
CA-3S	–	–	
GCA-3S	0.01 (0.00–0.01)	0.00 (0.00–0.00)	0.04
TCA-3S	–	–	
GCDCA	0.00 (0.00–0.00)	0.00 (0.00–0.00)	0.35
TCDCA	0.00 (0.00–0.00)	0.00 (0.00–0.00)	0.65
CDCA-3S	0.00 (0.00–0.00)	0.00 (0.00–0.00)	0.27
GCDCA-3S	0.07 (0.01–0.12)	0.01 (0.00–0.02)	0.06
TCDCA-3S	0.00 (0.00–0.01)	0.00 (0.00–0.00)	0.08
Others^a^	0.15 (0.11–0.21)	0.12 (0.08–0.15)	0.38

Values are presented as the median; values in brackets represent the interquartile range (IQR).

*UDCA* ursodeoxycholic acid, *CA* cholic acid, *CDCA* chenodeoxycholic acid, *GCA* glycocholic acid, *TCA* taurocholic acid, *CA-3S* cholic acid 3-sulfate, *GCA-3S* glycocholic acid 3-sulfate, *TCA-3S* taurocholic acid 3-sulfate, *GCDCA* glycochenodeoxycholic acid, *TCDCA* taurochenodeoxycholic acid, *CDCA-3S* chenodeoxycholic acid 3-sulfate, *GCDCA-3S* glycochenodeoxycholic acid 3-sulfate, *TCDCA-3S* taurochenodeoxycholic acid 3-sulfate.

^a^Others include hydroxylated bile acids, short-chain bile acids, and enteric bacterial producing bile acids.

### The correlation between serum bile acid profiles and comprehensive liver fibrosis markers

**Figure 3:**
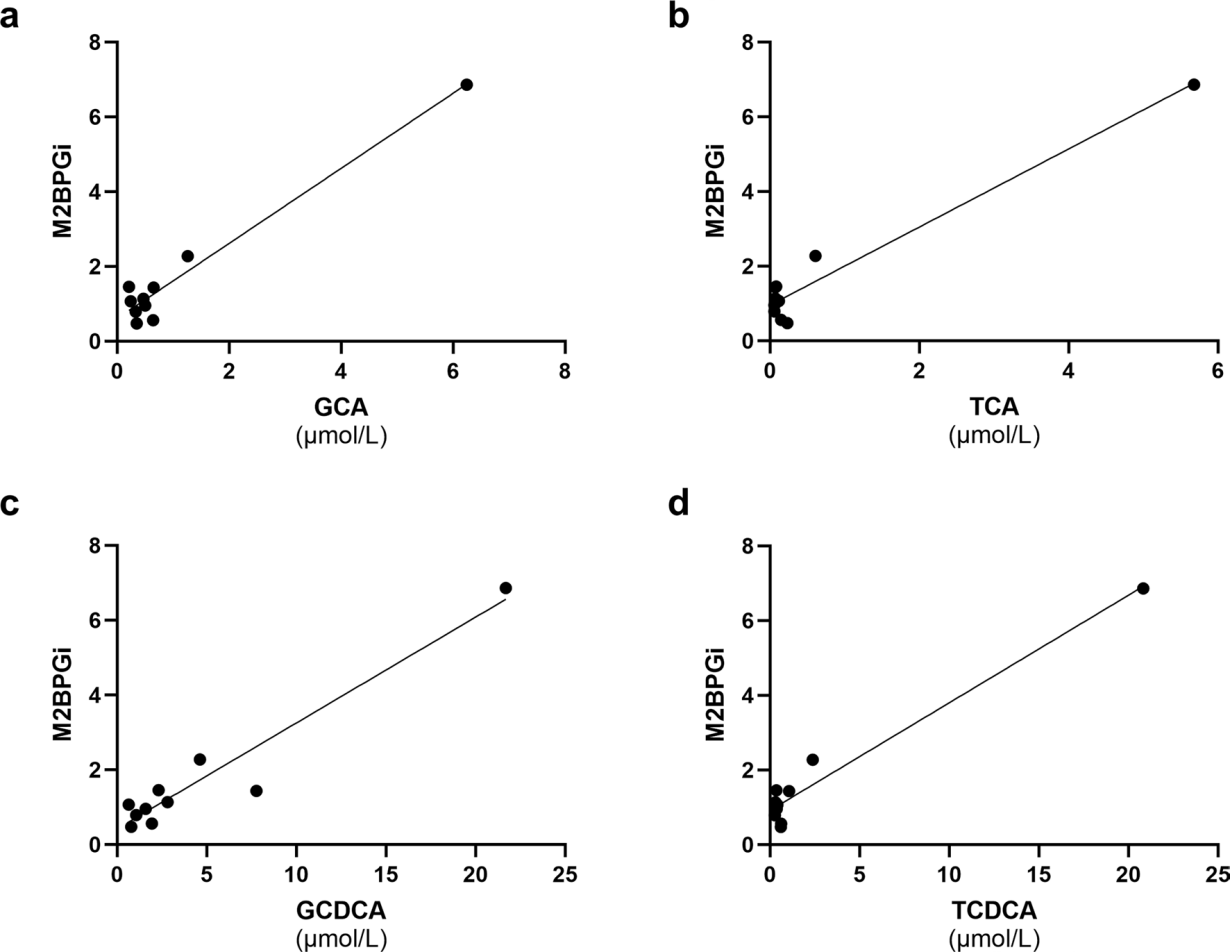
Correlations between serum glucuronide-conjugated CA, CDCA, and M2BPGi in patients with biliary atresia. There were no significant correlations between GCA and M2BPGi (Fig. 3a,  *r* = 0.41, *P* = 0.25) or TCA and M2BPGi (Fig. 3b, *r* = 0.32*, P* = 0.36). GCDCA was significantly correlated with M2BPGi (Fig. 3c, *r* = 0.78, *P* = 0.01), whereas TCDCA was not significantly correlated with M2BPGi (Fig. 3d, *r* = 0.42, *P* = 0.23).* CA* cholic acid, *CDCA* chenodeoxycholic acid, *M2BPGi* Mac-2 binding protein glycosylation isomer, *GCA* glycocholic acid, *TCA* taurocholic acid, *GCDCA* glycochenodeoxycholic acid, *TCDCA* taurochenodeoxycholic acid.

Serum glucuronide-conjugated bile acids, which were significantly higher in patients with biliary atresia, were used to assess the correlation between bile acid profiles and liver fibrosis markers. GCDCA showed a strong positive correlation with the Mac-2 binding protein glycosylation isomer (M2BPGi) (*r* = 0.78, *P* = 0.01) (Fig. 3). The levels of other bile acid compositions, including GCA, TCA, and TCDCA, showed no correlation with the Child–Pugh score, Model for End-Stage Liver Disease (MELD) score, or M2BPGi.

## Discussion

Analysis of the bile acid profiles of adult patients with biliary atresia with normalized bilirubin levels indicated that the slightly congested bile acid profiles were glucuronide-conjugated in serum and sulfate-conjugated in urine, suggesting the presence of a compensatory mechanism at work in mitigating bile acid hepatotoxicity, similar to mild liver cirrhosis. These bile acid congestion profiles closely resembled the patterns observed before Kasai portoenterostomy in patients with biliary atresia, as well as in patients with normalized bilirubin levels at 6 months after Kasai portoenterostomy in previous studies^2,10^. It provides valuable data indicating that bilirubin normalization after Kasai portoenterostomy does not lead to complete improvement in the excretion and metabolism of bile acids for more than 20 years. The typical characteristics of advanced cirrhosis, such as decreased serum CA to CDCA ratios, decreased serum glycine-to-taurine-conjugated ratios, and elevated urinary total and primary bile acids, were not observed^10–12^. High bile acid levels in biliary atresia, even after normalization of bilirubin levels, have been attributed to the destruction of the small bile ducts^13^. Prolonged impairment of bile acid transport proteins, such as sodium-taurocholate cotransporting polypeptide (NTCP) and organic solute transporter (OST) α/β, could also explain why serum bile acid levels can remain elevated and fluctuate after the improvement of cholestasis^2^. Based on these results, we believe it is worthwhile to pursue additional validation through a future genome-wide association study, exploring single nucleotide polymorphisms in hepatic bile acid transporters and bile acid synthesis enzymes in the context of biliary atresia.

A correlation between serum levels of GCDCA and M2BPGi, a non-invasive marker for hepatic fibrosis, was observed in patients with biliary atresia. In a recent report, serum levels of GCDCA and TCDCA were significantly correlated with portal pressure and showed a high potential to predict clinically significant portal hypertension in adults with cirrhosis^8^. In the early stages of cholestasis and cirrhosis, GCDCA was relatively higher than TCDCA^7^. Therefore, it is possible that only GCDCA was correlated with M2BPGi in this study, because adult patients with biliary atresia with normalized bilirubin levels had only slight bile acid congestion. Recently, M2BPGi has been shown to be sensitive to the degree of liver inflammation^14^. Whether bile acids themselves are correlated with the degree of liver fibrosis or potentially contribute to liver damage remains controversial^2^. In basic research using a cholestatic liver model, liver fibrosis and excess hepatic collagen deposition induced by GCDCA^15^, along with another report demonstrating its cytotoxic effects^16^. Ongoing clinical trials are expected to clarify the role of bile acids in biliary atresia^2,4^. We were unable to provide histological data on the liver tissue and stiffness using elastography. Therefore, our data were insufficient to demonstrate a clear correlation between bile acid toxicity, clinical abnormalities, and long-term liver fibrosis in biliary atresia. However, considering that the aim of our secondary objective was to present preliminary data suggesting an association between bile acid profiles and liver fibrosis, we believe that this objective was accomplished using a non-invasive liver fibrosis marker.

While the levels of serum secondary bile acids in patients with biliary atresia before Kasai portoenterostomy at 2 months of age were not significantly different from those in healthy controls^10^, the significantly lower levels observed in adults with biliary atresia suggests the possibility of impaired secondary bile acid production within the gut microbiota. After 2 years of age, secondary bile acid levels are reported to increase significantly in healthy subjects, ranging from 6% to one-third of total bile acids^9^. It is interesting to note that among our patients, five (50.0%) had cholangitis by 3 years of age, when the gut microbiota is usually considered to be established^17^; however, the levels of secondary bile acids were low in all patients, regardless of their history of cholangitis (Supplementary Table 3). Thus, the deficiency of secondary bile acids in biliary atresia may be affected by changes in the gut microbiota due to temporary cholestasis in infancy, perioperative antibiotics, or the original impairment of the bile acid synthesis process, rather than the use of antibiotics for repeated cholangitis^2^. Investigation of the gut microbiota and bile acid compositions from fecal samples of adult patients with biliary atresia has the potential to provide more information.

The present study was associated with several additional limitations, including the small sample size and lack of uniformity in conditions due to the administration of UDCA. However, it is essential to note that the number of adult patients with biliary atresia who have normalized bilirubin levels and normal liver function is limited. We collected data from 10 of 126 patients (7.9%) in our facility, which we believe is valuable for analyzing the physiological condition of the bile acid compositions in biliary atresia. Additionally, the effect of UDCA appeared to be limited, as there were no significant differences in other bile acid profiles in the subgroup analysis (Supplementary Table 2). Following oral administration, UDCA is primarily metabolized by the liver, mainly undergoing glycine conjugation to form glyco-UDCA (GUDCA) before being excreted into the bile or eliminated in the urine as sulfate-conjugated GUDCA^18^. Based on these findings, we concluded that the observed significant data were highly unlikely to be influenced by UDCA.

## Conclusion

In patients with biliary atresia, subclinical slight bile acid congestion persists into adulthood even after cholestasis has completely improved after Kasai portoenterostomy. These fundamental data on bile acid profiles indicate the value of further investigation as an etiological or therapeutic target for biliary atresia.

## Methods

### Study design and ethics

This was a prospective, single-center, observational study. Written informed consent was obtained from all enrolled patients as well as from healthy adult volunteers. This study was approved by the Juntendo University School of Medicine Institutional Review Board and complied with the 2013 Helsinki Declaration (Institutional Review Board number: E22-0011).

### Subjects, definitions, and criteria

Between January 1989 and December 2022, 126 patients with biliary atresia underwent Kasai portoenterostomy at our facility. Our native liver survival rates were 52.1% at 10 years and 46.0% at 20 years after Kasai portoenterostomy^19^, which is comparable to the average in Japan^20^. Focusing on adult native liver survivors (individuals aged 18 years and above), we excluded 63 patients with less than 18 years of follow-up, 1 patient who died from a cause unrelated to biliary atresia, and 9 patients who were lost to follow-up. Of the remaining 53 adult patients, we further excluded 23 patients who underwent liver transplantation, 5 patients who died, 2 patients with cystic type, and 12 patients with elevated total bilirubin levels exceeding 1.2 mg/dL or abnormal liver function, defined as levels of aspartate aminotransferase (AST) and alanine aminotransferase (ALT) exceeding 40 U/L. Among the remaining 11 patients, one patient who declined to participate in the study was excluded, leaving 10 of 126 patients (7.9%) with isolated biliary atresia for the evaluation of bile acid compositions.

The details of the ten patients with isolated biliary atresia are listed in Table 4. All the patients were diagnosed with cholangiography and liver biopsy. Kasai portoenterostomy techniques, pre-operative management, and post-operative protocols were consistent in all patients. The standard postoperative management protocols for antibiotics, choleretics, and corticosteroids have been previously reported^21^.

**Table 4:** Subjects in the perioperative period of portoenterostomy.

	Biliary atresia patients
The age at portoenterostomy (days)	53 (41–79)
Other abnormalities (n, %)	1 (10.0)
Jaundice clearance duration from portoenterostomy (days)	46 (39–55)
Cholangitis up to age 3 (n, %)	5 (50.0)
The number of episodes of cholangitis up to age 3 (n=5)	2 (1–2)

Values are presented as the median; values in brackets represent the interquartile range (IQR).

Two patients developed cholangitis after adulthood, but none developed cholangitis within 1 year of the bile acid analysis. Five of the ten patients continued to take UDCA at 600 mg/day and did not discontinue the medication; therefore, UDCA was excluded from the evaluation of the bile acid profiles. None of the patients was taking any other oral medications.

The bile acid profiles were compared and verified against samples obtained from healthy adult controls under the same conditions. The healthy individuals selected for the study had no history of significant illnesses or antibiotic treatment during infancy, and none had notable obesity. To verify the correlation between bile acid profiles and the degree of liver fibrosis, we examined the correlation between bile acid profiles and the Child–Pugh score, MELD score, and M2BPGi^22^. Ultrasonographic features refer to the ultrasonographic scoring system in the prediction of cirrhosis^23^.

### Serum and urinary sample preparation

The bile acid compositions were simultaneously verified in both serum and urine samples. To collect adequate samples, the fasting period was at least 6 h, and subjects were not allowed to eat or drink after dinner on the night before measurement. The collected blood was centrifuged at 3000 rpm for 5 min to obtain serum, which was then promptly frozen and stored at − 80 degrees Celsius. Urine samples were frozen immediately after collection and stored at − 80°C.

### Serum and urinary bile acid analysis by liquid chromatography-mass spectrometry (LS/MS)

After thawing, 10 μL of the internal standard was added to 50 μL of serum or urine samples. The solution was quickly transferred onto a solid-phase extraction cartridge (InertSep C18-B 100 mg/1 mL; Agilent Technologies Japan, Tokyo) that had been pre-conditioned with 1 mL of methanol and 3 mL of H_2_O. After loading the sample, the column was washed with 1 mL of H_2_O, and then the desired bile acids were eluted with 1 mL of 90% ethanol. After evaporation of the solvent using a centrifugal concentrator, the residue was dissolved in 0.2 mL of 50% ethanol, and then 5 μL of the solution was immediately analyzed by LC/MS. The methods used to analyze serum and urine bile acids by LC/MS were described in our previous studies^24,25^.

## Statistical analysis

The statistical analysis of differences between patients with biliary atresia and healthy controls was performed using the Fisher's exact test and the Mann–Whitney U test with GraphPad Prism, version 10.0.2. Correlations between bile acid profiles and liver fibrosis markers were analyzed using the Spearman's rho calculator. Statistical significance was defined as *P* < 0.05.

### Supplementary Information

Supplementary Table 1.

Supplementary Table 2.

Supplementary Table 3.

Supplementary Table 4.

Supplementary Figure 1.

## Data Availability

The datasets used and/or analyzed during the current study are available from the corresponding author on reasonable request.

## References

[1] Kasahara M (2019). Waiting list mortality for pediatric deceased donor liver transplantation in a Japanese living-donor-dominant program. Pediatr. Transpl..

[2] Harpavat S (2023). Serum bile acids as a prognostic biomarker in biliary atresia following Kasai portoenterostomy. Hepatology.

[3] Virk MK (2023). Elevated bile acids are associated with left ventricular structural changes in biliary atresia. Hepatol. Commun..

[4] Davenport M (2022). Surgical and medical aspects of the initial treatment of biliary atresia: Position paper. J. Clin. Med..

[5] Karpen SJ, Kelly D, Mack C, Stein P (2020). Ileal bile acid transporter inhibition as an anticholestatic therapeutic target in biliary atresia and other cholestatic disorders. Hepatol. Int..

[6] Laue T, Baumann U (2022). Odevixibat: an investigational inhibitor of the ileal bile acid transporter (IBAT) for the treatment of biliary atresia. Expert Opin. Investig. Drugs..

[7] Liu N (2019). Role of bile acids in the diagnosis and progression of liver cirrhosis: A prospective observational study. Exp. Ther. Med..

[8] Žížalová K (2023). Serum concentration of taurochenodeoxycholic acid predicts clinically significant portal hypertension. Liver Int..

[9] Jahnel J (2015). Reference ranges of serum bile acids in children and adolescents. Clin. Chem. Lab. Med..

[10] Golden J (2018). Liquid chromatography-mass spectroscopy in the diagnosis of biliary atresia in children with hyperbilirubinemia. J. Surg. Res..

[11] Sauerbruch T, Hennenberg M, Trebicka J, Beuers U (2021). Bile acids, liver cirrhosis, and extrahepatic vascular dysfunction. Front. Physiol..

[12] Suzuki M (2011). Urinary sulfated bile acid analysis for the early detection of biliary atresia in infants. Pediatr. Int..

[13] Nyholm I (2023). Serum FGF19 predicts outcomes of Kasai portoenterostomy in biliary atresia. Hepatology.

[14] Kimura Y (2021). Utility of mac-2 binding protein glycosylation isomer to evaluate graft status after liver transplantation. Liver Transpl..

[15] Hohenester S (2020). Glycochenodeoxycholate promotes liver fibrosis in mice with hepatocellular cholestasis. Cells.

[16] González-Rubio S (2015). GCDCA down-regulates gene expression by increasing Sp1 binding to the NOS-3 promoter in an oxidative stress dependent manner. Biochem. Pharmacol..

[17] Akagawa S, Kaneko K (2022). Gut microbiota and allergic diseases in children. Allergol. Int..

[18] Nagamatsu S (1997). Phase 1 clinical study of ursodesoxycholic acid (UR-PBC). Jpn. Pharmacol. Ther..

[19] Tsuboi K (2022). Native liver survivors of portoenterostomy for biliary atresia with excellent outcome: Redefining successful portoenterostomy. Pediatr. Surg. Int..

[20] Nio M (2017). Japanese biliary atresia registry. Pediatr. Surg. Int..

[21] Kobayashi H (2005). Optimum prednisolone usage in patients with biliary atresia postportoenterostomy. J. Pediatr. Surg..

[22] Shirabe K (2018). Mac-2 binding protein glycan isomer (M2BPGi) is a new serum biomarker for assessing liver fibrosis: More than a biomarker of liver fibrosis. J. Gastroenterol..

[23] Moon KM (2013). Ultrasonographic scoring system score versus liver stiffness measurement in prediction of cirrhosis. Clin. Mol. Hepatol..

[24] Sato K (2020). Changes in conjugated urinary bile acids across age groups. Steroids.

[25] Takei H (2022). Characterization of long-chain fatty acid-linked bile acids: A major conjugation form of 3β-hydroxy bile acids in feces. J. Lipid Res..

