# Supplementary material for: Bile acid profiles in adult patients with biliary atresia who achieve native liver survival after portoenterostomy
Source: Sci Rep. 2024 Jan 30;14:2492. doi: 10.1038/s41598-024-52969-6 (PMC10827714; doi:10.1038/s41598-024-52969-6)
Supplement: Supplementary file 1 — Supplementary Table 1. [file 41598_2024_52969_MOESM1_ESM.docx]

**Supplementary Table 1. Serum sulfated bile acids in each group.**

| Bile acids species | Biliary atresia patients | Healthy controls | *P* value |
| --- | --- | --- | --- |
| Primary (μmol/L) |  |  |  |
| CA-3S | - | - |  |
| GCA-3S | - | - |  |
| TCA-3S | - | - |  |
| CDCA-3S | - | - |  |
| GCDCA-3S | 0.00 (0.00-0.01) | 0.00 (0.00-0.00) | 0.38 |
| TCDCA-3S | 0.00 (0.00-0.00) | 0.00 (0.00-0.00) | 0.73 |
| Secondary (μmol/L) |  |  |  |
| DCA-3S | - | - |  |
| GDCA-3S | 0.00 (0.00-0.02) | 0.00 (0.00-0.02) | 0.85 |
| TDCA-3S | - | - |  |
| LCA-3S | - | - |  |
| GLCA-3S | - | - |  |
| TLCA-3S | - | - |  |

Values are presented as the median; values in brackets represent the interquartile range (IQR).

CA-3S: cholic acid 3-sulfate, GCA-3S: glycocholic acid 3-sulfate, TCA-3S: taurocholic acid 3-sulfate, CDCA-3S: chenodeoxycholic acid 3-sulfate, GCDCA-3S: glycochenodeoxycholic acid 3-sulfate, TCDCA-3S: taurochenodeoxycholic acid 3-sulfate, DCA-3S: deoxycholic acid 3-sulfate, GDCA-3S: glycodeoxycholic acid 3-sulfate, TDCA-3S: taurodeoxycholic acid 3-sulfate, LCA-3S: lithocholic acid 3-sulfate, GLCA-3S: glycolithocholic acid 3-sulfate, TLCA-3S: taurolithocholic acid 3-sulfate
