# Supplementary material for: Bile acid profiles in adult patients with biliary atresia who achieve native liver survival after portoenterostomy
Source: Sci Rep. 2024 Jan 30;14:2492. doi: 10.1038/s41598-024-52969-6 (PMC10827714; doi:10.1038/s41598-024-52969-6)
Supplement: Supplementary file 2 — Supplementary Table 2. [file 41598_2024_52969_MOESM2_ESM.docx]

**Supplementary Table 2.**

**Serum bile acid profiles in biliary atresia patients with and without ursodeoxycholic acid administration.**

| Bile acid species | Biliary atresia  with UDCA (n=5) | Biliary atresia  without UDCA (n=5) | *P* value |
| --- | --- | --- | --- |
| Total (μmol/L) | 8.28 (8.19-25.93) | 5.72 (4.60-7.01) | 0.14 |
| Total excluding UDCA (μmol/L) | 6.14 (3.77-12.19) | 4.47 (4.04-5.66) | 0.83 |
| Primary |  |  |  |
| Unconjugated (μmol/L) |  |  |  |
| CA | 0.05 (0.02-0.09) | 0.09 (0.04-0.09) | 0.67 |
| CDCA | 0.18 (0.05-0.55) | 0.20 (0.13-0.38) | 0.83 |
| Conjugated (μmol/L) |  |  |  |
| GCA | 0.50 (0.35-0.65) | 0.47 (0.24-0.64) | 0.67 |
| TCA | 0.07 (0.06-0.23) | 0.12 (0.08-0.15) | 0.47 |
| GCDCA | 1.59 (1.07-4.62) | 2.31 (1.94-2.82) | 0.83 |
| TCDCA | 0.61 (0.37-1.06) | 0.41 (0.35-0.61) | 0.92 |
| Ratios of CA to CDCA | 0.29 (0.27-0.29) | 0.28 (0.17-0.31) | 1 |
| Ratios of glycine-to-taurine-conjugated |  |  |  |
| CA | 5.50 (2.07-8.33) | 2.63 (2.00-4.27) | 0.40 |
| CDCA | 3.82 (1.93-4.30) | 3.18 (1.56-6.60) | 1 |
| Secondary |  |  |  |
| Unconjugated (μmol/L) |  |  |  |
| DCA | 0.03 (0-0.06) | 0.13 (0-0.15) | 0.40 |
| LCA | - | - |  |
| HCA | - | - |  |
| Conjugated (μmol/L) |  |  |  |
| GDCA | 0.06 (0-0.26) | 0.09 (0-0.16) | 1 |
| TDCA | 0.02 (0-0.08) | 0.04 (0-0.11) | 0.83 |
| GLCA | - | - |  |
| TLCA | - | - |  |
| GHCA | 0.00 (0-0.01) | 0.02 (0.01-0.04) | 0.14 |
| THCA | - | - |  |

Values are presented as the median; values in brackets represent the interquartile range (IQR).

UDCA: ursodeoxycholic acid, CA: cholic acid, CDCA: chenodeoxycholic acid, GCA: glycocholic acid, TCA: taurocholic acid, GCDCA: glycochenodeoxycholic acid, TCDCA: taurochenodeoxycholic acid, DCA: deoxycholic acid, LCA: lithocholic acid, HCA: hyocholic acid, GDCA: glycodeoxycholic acid, TDCA: taurodeoxycholic acid, GLCA: glycolithocholic acid, TLCA: taurolithocholic acid, GHCA: glycohyocholic acid, THCA: taurohyocholic acid

Serum sulfate-conjugated bile acids were extremely low in both groups and were omitted from the analysis.
