# Supplementary material for: Bile acid profiles in adult patients with biliary atresia who achieve native liver survival after portoenterostomy
Source: Sci Rep. 2024 Jan 30;14:2492. doi: 10.1038/s41598-024-52969-6 (PMC10827714; doi:10.1038/s41598-024-52969-6)
Supplement: Supplementary file 3 — Supplementary Table 3. [file 41598_2024_52969_MOESM3_ESM.docx]

**Supplementary Table 3. Serum bile acid profiles in biliary atresia with and without cholangitis.**

| Bile acid species | Biliary atresia  with cholangitis (n=5) | Biliary atresia  without cholangitis (n=5) | *P* value |
| --- | --- | --- | --- |
| Total (μmol/L) | 8.19 (5.72-8.28) | 7.32 (7.01-27.9) | 0.67 |
| Total excluding UDCA (μmol/L) | 4.47 (2.64-6.14) | 5.66 (4.04-17.83) | 0.40 |
| Primary |  |  |  |
| Unconjugated (μmol/L) |  |  |  |
| CA | 0.04 (0.02-0.13) | 0.09 (0.05-0.09) | 0.83 |
| CDCA | 0.10 (0.05-0.13) | 0.38 (0.20-0.65) | 0.06 |
| Conjugated (μmol/L) |  |  |  |
| GCA | 0.35 (0.33-0.64) | 0.50 (0.47-0.65) | 0.67 |
| TCA | 0.15 (0.12-0.23) | 0.07 (0.07-0.08) | 0.46 |
| GCDCA | 1.07 (0.78-1.94) | 2.82 (2.31-7.77) | 0.09 |
| TCDCA | 0.61 (0.41-0.61) | 0.37 (0.35-1.06) | 1 |
| Ratios of CA to CDCA | 0.31 (0.29-0.34) | 0.17 (0.11-0.28) | 0.04 |
| Ratios of glycine-to-taurine-conjugated |  |  |  |
| CA | 2.07 (2.00-4.23) | 6.71 (2.62-8.33) | 0.30 |
| CDCA | 1.93 (1.56-3.18) | 6.60 (4.30-7.33) | 0.14 |
| Secondary |  |  |  |
| Unconjugated (μmol/L) |  |  |  |
| DCA | 0.10 (0.06-0.15) | 0.00 (0-0.03) | 0.17 |
| LCA | - | - |  |
| HCA | - | - |  |
| Conjugated (μmol/L) |  |  |  |
| GDCA | 0.16 (0.09-0.26) | 0.00 (0-0.06) | 0.35 |
| TDCA | 0.08 (0.04-0.11) | 0.00 (0-0.02) | 0.35 |
| GLCA | - | - |  |
| TLCA | - | - |  |
| GHCA | 0.00 (0-0.01) | 0.04 (0.02-0.07) | 0.21 |
| THCA | - | - |  |

Values are presented as the median; values in brackets represent the interquartile range (IQR).

UDCA: ursodeoxycholic acid, CA: cholic acid, CDCA: chenodeoxycholic acid, GCA: glycocholic acid, TCA: taurocholic acid, GCDCA: glycochenodeoxycholic acid, TCDCA: taurochenodeoxycholic acid, DCA: deoxycholic acid, LCA: lithocholic acid, HCA: hyocholic acid, GDCA: glycodeoxycholic acid, TDCA: taurodeoxycholic acid, GLCA: glycolithocholic acid, TLCA: taurolithocholic acid, GHCA: glycohyocholic acid, THCA: taurohyocholic acid

Serum sulfate-conjugated bile acids were extremely low in both groups and were omitted from the analysis.
