# Supplementary material for: Bile acid profiles in adult patients with biliary atresia who achieve native liver survival after portoenterostomy
Source: Sci Rep. 2024 Jan 30;14:2492. doi: 10.1038/s41598-024-52969-6 (PMC10827714; doi:10.1038/s41598-024-52969-6)
Supplement: Supplementary file 4 — Supplementary Table 4. [file 41598_2024_52969_MOESM4_ESM.docx]

**Supplementary Table 4. Urinary secondary bile acids in each group.**

| Bile acids species | Biliary atresia patients | Healthy controls | *P* value |
| --- | --- | --- | --- |
| Secondary |  |  |  |
| Unconjugated (μmol/L) |  |  |  |
| DCA | 0.00 (0.00-0.00) | 0.00 (0.00-0.00) | 0.43 |
| LCA | - | - | - |
| HCA | - | - | - |
| Conjugated (μmol/L) |  |  |  |
| GDCA | 0.00 (0.00-0.00) | 0.00 (0.00-0.00) | 0.21 |
| TDCA | - | - | - |
| DCA-3S | 0.00 (0.00-0.00) | 0.00 (0.00-0.00) | 0.73 |
| GDCA-3S | 0.03 (0.01-0.13) | 0.01 (0.00-0.12) | 0.55 |
| TDCA-3S | 0.00 (0.00-0.00) | 0.00 (0.00-0.01) | 0.79 |
| GLCA | - | - | - |
| TLCA | - | - | - |
| LCA-3S | - | - | - |
| GLCA-3S | 0.00 (0.00-0.02) | 0.00 (0.00-0.03) | 0.88 |
| TLCA-3S | 0.00 (0.00-0.00) | 0.00 (0.00-0.01) | 0.97 |
| GHCA | 0.00 (0.00-0.01) | 0.00 (0.00-0.00) | 0.12 |
| THCA | - | - | - |

Values are presented as the median; values in brackets represent the interquartile range (IQR).

DCA: deoxycholic acid, LCA: lithocholic acid, HCA: hyocholic acid, GDCA: glycodeoxycholic acid, TDCA: taurodeoxycholic acid, DCA-3S: deoxycholic acid 3-sulfate, GDCA-3S: glycodeoxycholic acid 3-sulfate, TDCA-3S: taurodeoxycholic acid 3-sulfate, GLCA: glycolithocholic acid, TLCA: taurolithocholic acid, LCA-3S: lithocholic acid 3-sulfate, GLCA-3S: glycolithocholic acid 3-sulfate, TLCA-3S: taurolithocholic acid 3-sulfate, GHCA: glycohyocholic acid, THCA: taurohyocholic acid
