# Supplementary figures and images for: Bile acid profiles in adult patients with biliary atresia who achieve native liver survival after portoenterostomy
Source: Sci Rep. 2024 Jan 30;14:2492. doi: 10.1038/s41598-024-52969-6 (PMC10827714; doi:10.1038/s41598-024-52969-6)

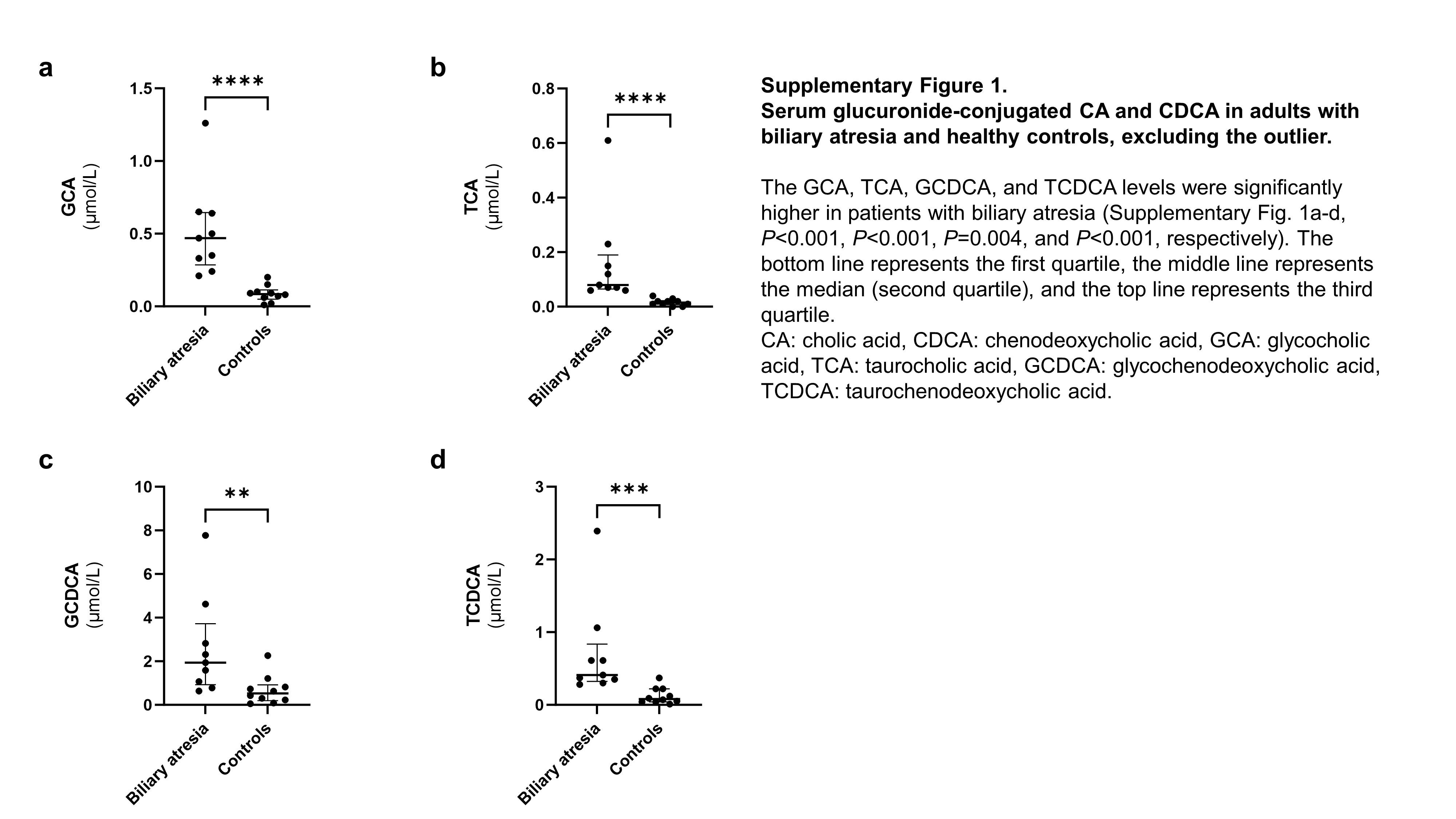

Supplement: Supplementary file 5 — Supplementary Figure 1. [file 41598_2024_52969_MOESM5_ESM.tif]
